# Characterizing health researcher barriers to sharing results with study participants

**DOI:** 10.1017/cts.2019.409

**Published:** 2019-10-04

**Authors:** Pearl A. McElfish, Christopher R. Long, Laura P. James, Aaron J. Scott, Elizabeth Flood-Grady, Kim S. Kimminau, Robert L. Rhyne, Mark R. Burge, Rachel S. Purvis

**Affiliations:** 1 Internal Medicine, College of Medicine, University of Arkansas for Medical Sciences Northwest, Fayetteville, AR 72703, USA; 2 Department of Pediatrics, University of Arkansas for Medical Sciences, Little Rock, AR 72205, USA; 3 Office of Community Health and Research, University of Arkansas for Medical Sciences Northwest, Fayetteville, AR 72703, USA; 4 STEM Translational Communication Center, College of Journalism and Communications and Recruitment Center, Clinical Translational Science Institute, University of Florida, Gainesville, FL 32611, USA; 5 Department of Family Medicine and Community Health, University of Kansas Medical Center, Kansas City, KS 66160, USA; 6 Department of Family and Community Medicine, School of Medicine, University of New Mexico, Albuquerque, NM 87131, USA; 7 Clinical and Translational Science Center, Health Science Center, University of New Mexico, Albuquerque, NM 87131, USA

**Keywords:** Dissemination, results sharing, research communication, engagement, barriers

## Abstract

**Introduction:**

Research participants want to receive results from studies in which they participate. However, health researchers rarely share the results of their studies beyond scientific publication. Little is known about the barriers researchers face in returning study results to participants.

**Methods:**

Using a mixed-methods design, health researchers (*N* = 414) from more than 40 US universities were asked about barriers to providing results to participants. Respondents were recruited from universities with Clinical and Translational Science Award programs and Prevention Research Centers.

**Results:**

Respondents reported the percent of their research where they experienced each of the four barriers to disseminating results to participants: logistical/methodological, financial, systems, and regulatory. A fifth barrier, investigator capacity, emerged from data analysis. Training for research faculty and staff, promotion and tenure incentives, and funding agencies supporting dissemination of results to participants were solutions offered to overcoming barriers.

**Conclusions:**

Study findings add to literature on research dissemination by documenting health researchers’ perceived barriers to sharing study results with participants. Implications for policy and practice suggest that additional resources and training could help reduce dissemination barriers and increase the return of results to participants.

## Background/Introduction

Participants want to receive study updates and results from the research in which they participate, [1–7] and researchers generally support the concept of returning results to study participants [8–13]. However, most researchers report that they do not return results to participants [7,9,11,12]. Disseminating study results beyond scientific publication can raise public awareness about the importance of research and increase trust in the research process among current and prospective participants [10]. Incongruence between participant expectations for receiving study results and researcher practices for disseminating study findings may deter individuals from participating in future studies [6]. Thus, there is a need to investigate the reasons health researchers are not sharing results with study participants.

There is limited research examining why researchers do not disseminate (i.e., actively return, share) results to study participants. A 2013 Agency for Healthcare Research and Quality (AHRQ) report identified significant gaps in knowledge of research dissemination best practices [14]. The few published studies have sought to understand researchers’ barriers to broad public dissemination practices [15], or have focused on specific health conditions (e.g., cancer) [11,16], rather than investigating researcher’s perceived barriers to returning study results to research participants. Understanding the barriers to sharing results with participants from the perspective of health researchers is necessary to bridge the gap between participant’s expectations for receiving study results and researcher’s interest in returning results to study participants. To address this important gap in knowledge, this study examined health researchers’ perceived barriers to returning study results to particpants i.e., participant-level dissemination. For the purpose of this paper, we define participant-level dissemination as communicating de-identified, aggregate information about study findings to persons who participated in the study through means other than peer-reviewed publications.

## Method

A mixed-methods concurrent triangulation design [17–23], with a survey that collected quantitative and qualitative data simultaneously, assessed the perceptions and barriers health researchers experience with the dissemination of results to study participants. The study protocol and general findings are provided elsewhere [24,25]. This article focuses on an in-depth examination of the perceived barriers as they were described by survey respondents.

### Participant Recruitment

Respondents were aged 18 and older with a faculty or a postdoctoral appointment at a US academic medical institution. All self-reported to be health researchers who conduct research that requires the consent of human subjects. Efforts to recruit respondents were focused on universities with Clinical and Translational Science Award (CTSA) programs and Prevention Research Centers (PRCs) [26,27]. Initial recruitment contact was through Principal Investigators (PIs) at CTSAs and PRCs.

Initial electronic correspondence with CTSA and PRC PIs included a brief overview of the study and a survey invitation template that PIs could send to their institution’s human subjects research investigators. The e-mail template provided respondents with the opportunity to confirm eligibility and provide consent electronically. A second e-mail was sent 2 weeks after the initial contact asking the PIs at the CTSAs and PRCs to send out a reminder e-mail to their investigators. Some respondents also forwarded the survey to associates and collaborators who may not be part of a CTSA or PRC. All respondents who affirmed on the first page of the survey that they met the inclusion criteria were allowed to complete the survey. This project was determined to be exempt from human protections oversight by the IRB at the lead authors’ university (#205983).

### Data Capture

Respondents completed a mixed-method electronic survey via Research Electronic Data Capture (REDCap) [28]. Respondents were asked to broadly identify any barriers that they faced when returning results to participants. Then respondents were asked to use a slider scale to indicate the percentage of their studies where they encountered different types of barriers. Open-ended questions were used throughout the survey to encourage respondents to provide greater in-depth, qualitative responses to share experiences and provide examples. The average time for survey completion was less than 10 minutes.

### Analytic Strategy

Respondent charateristics and preceived prevalence of barriers to sharing results with study participants were calculated as descriptive summaries. Open-ended responses were coded for both a priori and emergent codes. A priori codes were selected from four areas identified in the previous literature [8–12,29,30]. These themes are: logistical/methodological, financial, systems, and regulatory. During analysis of open-ended responses, an additional theme emerged as “investigator capacity” barriers. The codes were defined, discussed, and incorporated into a detailed codebook. All qualitative data were subsequently coded based on the codebook themes. A coding template was used to organize the large amount of open-ended responses [31,32]. Three qualitative researchers critically reviewed coded data independently to ensure scientific rigor. Any differences in coding were discussed by the research team until they reached consensus. The most representive quotes for each barrier theme were selected to summarize the findings [33,34].

## Results

### Respondent Characteristics

Survey data colletion ran from March 8, 2017 to April 26, 2017. During this period, 414 respondents consented electronically and responded to at least 1 survey question, and 355 respondents completed the survey. Researchers from more than 40 universities participated. See Table 1 for respondents’ demographic characteristics. The mean age of respondents was 50.6 years; over half (56.4%) held a PhD, and most of the respondents had academic appointments in medicine (65.2%). Respondents reported conducting a mean of 14.0 health research studies as PI, Co-PI, or Co-Investigator.

**Table 1.** Characteristics of study respondents

	Number (% of survey respondents) or Mean ± SD
**Gender (*n* = 350)**	
Female	202 (57.7)
Male	147 (42.0)
Other	1 (0.3)
**Age (*n* = 311)**	50.6 ± 11.3
25–34	22 (7.1)
35–44	77 (24.8)
45–54	100 (32.2)
55–64	74 (23.8)
65 and older	38 (12.2)
**Degrees held (*n* = 353)**^a^	
PhD	199 (56.4)
MD	158 (44.8)
MPH	38 (10.8)
**Primary academic appointment (*n* = 359)**	
Medicine	234 (65.2)
Public Health	30 (8.4)
Allied Health Professions	24 (6.7)
Nursing	21 (5.8)
Other	50 (13.9)
**Ever served as PI, Co-PI, or Co-I for the following funders (*n* = 359)**^a^	
NIH	262 (73.0)
CDC	53 (14.8)
AHRQ	42 (11.7)
PCORI	39 (10.9)
Other	186 (51.8)

*Note.* Percentages are based on the number of valid responses for each item.

a Respondents could select more than one response. Table adapted from Long *et al*. [25].

Abbreviations: AHRQ, Agency for Healthcare Research and Quality; CDC, Centers for Disease Control; Co-I, co-investigator; Co-PI, co-principal investigator; NIH, National Institutes of Health; PCORI, Patient-Centered Outcomes Research; PI, principal investigator.

### Identifying Barriers

Four a priori themes were included in the slider scale questions: (1) logistical/methodological barriers, (2) financial barriers, (3) systems barriers, and (4) regulatory barriers, as depicted in Table 2. One additional theme that emerged from the qualitative data was (5) investigator capacity barriers, which was not included as a quantitative item. All five themes are described in the qualitative findings.

**Table 2.** Perceptions of barrier-specific prevalence to sharing results with study participants

	Number (% of survey respondents)
**For what proportion of your studies do you believe each of the following has been a barrier to sharing your results with research participants?**	
**Logistical/methodological barriers (*n* = 376)**	
0% of my studies	43 (11.4)
1–49% of my studies	126 (33.5)
50–100% of my studies	207 (55.1)
**Financial barriers (*n* = 378)**	
0% of my studies	52 (13.8)
1–49% of my studies	122 (32.3)
50–100% of my studies	204 (54.0)
**Systems barriers (*n* = 376)**	
0% of my studies	67 (17.8)
1–49% of my studies	107 (28.5)
50–100% of my studies	202 (53.7)
**Regulatory barriers (*n* = 378)**	
0% of my studies	60 (15.9)
1–49% of my studies	162 (42.9)
50–100% of my studies	156 (41.3)

*Note.* Percentages are based on the number of valid responses for each slider scale item, where possible responses ranged from 0 to 100%. Table adapted from Long *et al*. [25].

## Logistical/Methodological Barriers

On the slider scale, most researchers (88.6%) reported logistical/methodological barriers to returning results in at least 1% of their studies, with 55.1% reporting this domain in at least half of the studies they conducted. In the open-ended responses, respondents described a lack of: ability to contact participants; effective and efficient communication methods; and tools and training as the logistical/methodological barriers that precluded them from returning results to participants.

One of the most consistently referenced logistical/methodological barriers was the inability to recontact participants. “Once the study is over there is no easy mechanism to contact the participants” (ID#405). Many respondents stated that they “did not collect contact information about any of the participants, so we did not have a way to share results” (ID#532). The time lapse between recruiting participants, their study participation, and when data are analyzed and ready for dissemination represents a barrier to recontacting participants. Respondents discussed the difficulty this caused in sharing results. “Time lag. Often, it takes many months or even years to get results completed. By then, a substantial portion of participants’ contact information is no longer accurate” (ID#88). Other respondents echoed this experience: “By the time all analyses are conducted, many of our participants have been lost for contact or passed away” (ID#457) and “time is the number one barrier to dissemination, particularly given the usual long period of time between subject enrollment and publication” (ID#527).

The second most consistent logistical/methodological barrier respondents commented on was a paucity of methods for effective and efficient communication with participants. Respondents cited uncertainty and concerns about what method(s) of communication to use. One respondent succinctly summarized this barrier: “This is probably the biggest barrier. How do we effectively communicate to the participants? Do we use mail? Email? Bring all of the participants back in?” (ID#532). Other respondents cited the same concern regarding lack of communications methods. “Figuring out how to present the results ‐ paper format through the mail or email versus oral presentation. If you decide to do an oral presentation, there are a lot of logistics with planning that as well” (ID#32).

Respondents reported a lack of institutional support, tools, and training related to returning results to participants, “there is very little support at the institutional level for community dissemination” (ID#95), “lack of institutional support for investigator to ensure results are provided” (ID#509), and “infrastructure at one’s institution to actually facilitate this dissemination of aggregate results with the research study participants in a consistent way is a factor” (ID#399). Several respondents recommended that their university provide tools and training to help investigators return results to respondents. “It would be great to have training or resources…for example a webinar, brief workshop, or even a folder of shared examples of materials that I could use. By seeing what others have done, I could quickly adapt the ideas” (ID#18). Other respondents stated that they needed “training in semiotics and information design” (ID#233).

Even those respondents who had returned results of prior studies cited logistical/methodological concerns with which methods to use to share results with study participants. “For a different study where we did return summary results it was a huge effort to create a participant friendly result, organize mailing, update and maintain addresses, answer queries based on the results, and process returned mailings” (ID#4).

## Financial Barriers

On the slider scale, 86.2% of respondents reported financial barriers to returning study results in at least 1% of their studies, and 54.0% reported financial barriers in at least half of the studies they previously conducted. In the open-ended responses, respondents stated they did not have the financial resources to disseminate results to participants. “We have no funded time for this sort of activity” (ID#13), and “no funding to support dissemination of results to participants” (ID#500). Respondents also stated they believed “it’s too costly to share” results (ID#520), and “most studies do not include [the] cost of dissemination of results in their budget, and costs can be significant” (ID#95).

In addition to discussing a general lack of funding for dissemination, respondents described financial barriers such as a lack of money for mailings, postage, and meeting costs (e.g., space, parking, food) related to returning results to participants. Respondents described a “lack of funds to produce the newsletters, no money for mailing” (ID#273), and “funding needed to create dissemination materials” (ID#351). Respondents also discussed the lack of funding for returning results through community meetings, which would require travel, food, and other event costs: “Lack of funding to support ‘Report to the Community’ events and food to offer to attendees, which is customary in many cultures” (ID#519), “Best way to reach my participants is through community events, requiring support such as meals” (ID#693), and “Funding needed…travel expenses, and potential cost to host a community event to cover venue and food costs” (ID#351).

Respondents commented on a lack of funding to pay for the staff required to return results to participants. They explained that “one would need the budget to have support staff to carry out the task” (ID#528), “you do not have the funds to mail items nor sometimes funds to support the staff to do the mailings” (ID#301), and there is often “no money for staff support to compile distribution lists and send out results” (ID#589). Some respondents stated that if financial barriers were removed, then researchers would be more likely to disseminate results to participants. “I would suspect that if funding were available to support dissemination, that PIs would support…first-level dissemination” (ID#192).

## Systems Barriers

On the slider scale, 82.2% of respondents reported systems barriers in at least 1% of their studies, and 53.7% of respondents reported systems barriers in at least half of their previous studies. In the open-ended responses, there were two frequently stated systems barriers for disseminating research results to participants. The first included two components: a lack of promotion and tenure recognition/incentive and a lack of dissemination requirements from funders. When respondents cited the lack of incentive related to their academic promotion and tenure, they noted that “in academia you get tenure based solely on how many papers you publish and how many grants you get” (ID#37). Another respondent summarized “there is zero incentive to disseminating results to research participants and [it] takes away from your time to do the things that will get you tenure and promotion” (ID#37). Several respondents elaborated and explained that researchers faced several competing demands, and there was not a clear incentive to disseminate results to participants: “In the face of competing demands, faculty are unlikely to undertake extra tasks that are not associated with incentives (‘carrots’ or ‘sticks’)” (ID#18), and “investigators [are] frequently under more pressure to publish study results and obtain the future grant funding, and therefore, neglect to work at providing results to study participants” (ID#27). Others had similar responses. “Sharing results with participants is not emphasized as a critical piece of the research process…the focus of the researcher is publication through traditional academic pathways, and I think dissemination to the participants gets lost” (ID#526).

The second systems barrier most frequently mentioned was the lack of a requirement from funding agencies. Respondents cited that the NIH and other funding agencies often do not fund or prioritize dissemination, stating: “funding by NIH was not provided for dissemination of results to participants” (ID#79), and “frankly, spending money to be ‘good citizens’ doesn’t get your grant funded” (ID#10). Respondents stated that they perceived that returning results to participants was not a priority of funders. “Grant budgets are not increasing and the priority is on the science. If this [dissemination of research results to participants] is not required, then it will not be priority” (ID#14).

## Regulatory Barriers

On the slider scale, 84.1% of respondents reported regulatory barriers to returning results to participants in at least 1% of their studies, and 41.3% reported regulatory barriers in at least half of their previous studies. In the open-ended responses, respondents had the perception that “if you were to share such information, it would need to be IRB-regulated and approved” (ID#25). Respondents also perceived that it was “difficult to share the data without often violating IRB or HIPAA rules ‐ even aggregate data” (ID#334). Respondents perceived that returning results would increase the risk to participants’ privacy and “would require researchers to collect participant’s personal data which increases the risk from an IRB” (ID#597). Many respondents stated that their institution’s IRB should provide more guidance regarding returning results to participants: “the lack of an IRB approved mechanism or infrastructure at one’s institution to actually facilitate this dissemination of aggregate results with the research study participants in a consistent way is a factor” (ID#399), and “describing how results will be disseminated to participants should be a more important part of [the] IRB” (ID#359).

## Investigator Capacity Barriers

Investigator capacity barriers emerged in the qualitative data as a theme. Within the theme of investigator capacity barriers, several subthemes also emerged, including: investigator awareness, skill, and time related to participant dissemination. Investigator capacity barriers was not an a priori theme, and therefore, lacks the quantitative data to document the proportion of studies in which respondents reported investigator barriers. However, the vast majority of respondents discussed investigator capacity barriers in their qualitative response.

The most common investigator capacity barrier identified by respondents was a lack of awareness related to returning study results. Respondents stated “I simply didn’t consider it” (ID#566), “it honestly didn’t occur to me” (ID#622), “never occurred to me” (ID#478), and “I have never considered disseminating results of any of my studies” (ID#573). Some respondents even noted that they might consider disseminating results in the future, now that it had been brought to their attention through their participation in this study. “I didn’t consider this as an option, but I am interested in doing this for the future” (ID#96).

Another common investigator capacity barrier pertains to how scientists are trained, or in many cases, their lack of training. “We are trained to write scientific papers. It’s another skill set to write scientific results for a non-scientific audience” (ID #23). Respondents consistently stated researchers did not have the writing skills that would allow them to disseminate research results to participants: “As scientists, we are not trained to disseminate results to lay audiences. May be intimidating/difficult to do” (ID#37), “It takes … communication skills that many researchers were never taught and most do not have” (ID#401), and “scientific writing for publication is different from writing for the lay audience. The challenge lies in simplifying the results without diluting the message/findings of the study” (ID#56).

Respondents also stated that they did not have time to disseminate results to participants. “Lack of time with multiple competing demands is a major limitation of dissemination” (ID#306). Others identified that disseminating results to participants was not part of their research timeline. “There is not likely to be a specific time to share results built into the clinical trial model” (ID#412). Respondents discussed competing demands for their time that did not leave time for returning results to participants and stated: “Researchers are overwhelmed with obligations” (ID#412), “we all feel we have so much to do [and] finding time is a huge issue” (ID#169), and that “lack of time is probably the reason dissemination doesn’t routinely happen. It’s a terrible excuse, but unfortunately true” (ID#490).

## Discussion

Returning results to participants is an important part of translational research. Although research participants are interested in receiving the results from studies in which they participated and researchers generally agree on the importance of providing the results to participants, we know that study results are rarely returned to particpants. In an effort to bridge this gap, we examined health researchers’ perceived barriers to returning study results to participants. Four barriers were identified and respondents reported the percentage of their studies that encountered each of these barriers: (1) logistical/methodological barriers, (2) financial barriers, (3) systems barriers, and (4) regulatory barriers. An additional barrier, “investigator capacity” was identified during qualitative data analysis (See Table 3.)

**Table 3.** Health research dissemination challenges and recommendations

Challenges/Opportunities	Recommendations
Logistical/Methodological Lack of ability to contact participants Lack of effective and efficient communication methods Lack of tools and training from institution Financial Lack of funding for staff to return results to participants Lack of money for mailings and meeting-related expenses to return results to participants	Funding agencies: Require a dissemination plan that includes returning results to participants as part of funding applications Include funding to cover the cost of returning results to participants Acknowledge researchers who document prior efforts to return results to participants
Systems Lack of promotion and tenure recognition/incentive for efforts to return results to participants Lack of requirement from funder to return results to participants Regulatory Lack of understanding of IRB privacy protections related to returning results to participants Lack of guidance from IRB on how to return results to participants	CTSAs and PRCs Provide training to research faculty and staff on how to effectively return results to participants Develop and disseminate tools, templates, and protocols for returning results to participants Create dissemination courses that assist researchers in their efforts to return results to participants Encourage changes to promotion and tenure policies that provide recognition/incentives for returning results to participants
Investigator capacity Lack of investigator awareness that they should return results to participants Lack of investigator skill on how to return results to participants Lack of investigator time to return results to participants	IRBs Clarify privacy policies and how they affect returning results to participants Require plans for returning results to participants in research protocols Recommend or require options for receiving results as part of the consent process

Abbreviations: CTSA, Clinical and Translational Science Award; IRB, Institutional Review Board; PRC, Prevention Research Center.

Respondents discussed several logistical/methodological barriers including a lack of ability to contact participants, a lack of effective and efficient communication methods, and a lack of tools and training for disseminating findings at their institution. Similarly, respondents articulated how the lack of awareness, skill, and time precluded investigators from sharing results with study participants. These findings are consistent with Brownson *et al*. that public health faculty lack the tools and skills to communicate research effectively to lay public audiences [15]. Our study is the first to document this in a broad sample of researchers focused on returning results to study participants. It is also the first to document that respondents want training and tools to help them overcome dissemination barriers.

Universities, particularly those with CTSAs and PRCs, are well positioned to help researchers overcome these barriers. To overcome both logistical/methodological and investigators capacity barriers to dissemination, CTSAs and PRCs should develop and disseminate training for research faculty and staff that focuses on how to effectively return results to participants. CTSAs and PRCs should also develop and disseminate tools, templates, and protocols for returning results to participants. It may be most effective to create dissemination cores modeled after other research cores, to assist researchers in growing their skills and supporting the return of results to participants.

A second group of key findings pertains to the lack of promotion and tenure incentives and a lack of requirement from funding agencies to disseminate study results to participants. Respondents focused their efforts on activities (e.g., publishing, grant writing) that are recognized and rewarded through promotion and tenure and prioritized by funding agencies. Although faculty focus mostly on tasks related to promotion and tenure, and avoid tasks that do not count toward promotion and tenure or does not help them secure funding [35,36], identifying ways to acknowledge and reward dissemination to the lay public are important steps to increasing the return of results to participants.

To increase dissemination efforts and results sharing with study participants, CTSAs, PRCs, funding agencies, and academic institutions should encourage changes to promotion and tenure policies that recognize and value returning results to participants. Participant dissemination products and efforts should also be included on faculty *curricula vitae* and in promotion and tenure packets. Similarly, funding agencies could require a dissemination plan that addresses how investigators will return results to participants as part of grant applications and acknowledge researchers’ prior efforts to return results to participants in grant review processes. While several funding agencies have statements supporting dissemination of results to participants, few (e.g., Patient-Centered Outcomes Research Institute) require a dissemination plan that includes returning results to participants [37].

A lack of financial resources was cited as a significant barrier. This finding is consistent with Partridge *et al*. that documented cancer researchers’ concerns with the lack of resources to return results to participants as a primary barrier [11]. However, this is the first study to identify the barriers from a large sample of researchers not specific to cancer research, thereby filling an important gap in the current literature. While many funding agencies have begun to encourage dissemination of research findings to participants, most funders are not requiring or providing funding for the return of results to participants [37]. Public and private funders of research can do more to change policy and practice, and should provide funding to cover the cost of returning results to participants as part of the expected research costs.

One of the most surprising findings was that respondents perceived that IRBs constrain researchers from returning results to participants. Many respondents seemed to misunderstand privacy regulations and reported that the IRB would not approve the return of results to participants. On the contrary, many IRBs ask that researchers use the consent document to indicate how aggregate results will be shared with research participants [38–40]. Informing participants of dissemination plans in the consent document fulfills the ethical obligation researchers have to share study findings with research participants [10,39,40]. To address these misunderstandings, representatives from IRBs, as well as those responsible for regulating research conduct (e.g., Offices of Research and Privacy) should work with investigators to clarify privacy policies and how they relate to returning research results to participants. IRBs could also require plans for returning results to participants in research protocols and encourage researchers to provide an option for returning results to participants as part of the consent process.

### Strengths and Limitations

This study has some limitations. First, we were unable to calculate a response rate, because it is unknown how many eligible investigators were sent and received the e-mail invitation to participate in the survey. Second, while the sample size is large and included respondents from more than 40 research institutions, the responses may not be representative of all health researchers in the USA. Specifically, respondents who chose to complete the survey may have been biased by their interest in the topic.

Despite these limitations, this study has several strengths. First, while various entities (The Secretary’s Advisory Committee on Human Research Protections, The National Academies of Sciences, Engineering, and Medicine, and The Multi-Regional Clinical Trials Center) have produced reports on sharing results with research participants [41–43], their recommendations are focused most often on providing individual results. This is the first in-depth analysis of the barriers health researchers experience sharing aggregate study results with research participants. Thus, our findings elucidate specific reasons for the incongruence between researchers’ acknowledgment that participants should receive the results from studies in which they participate and the lack of dissemination of results to participants. Second, this article moves beyond recommendations of these agencies by identifying specific implementation strategies that could help research overcome barriers and improve the return of results to participants. Third, this study was strengthened by the mixed-method design. The mixed-methods approach – specifically, the qualitative data – allowed for the emergence of an additional theme (i.e., investigator capacity barriers), even though this domain was not included among the quantitative items. Most importantly, the study provides an important foundation for improving policies and practices that can increase the return of results to participants.

## Conclusion

Returning study results to participants is an important part of the translational research process. While researchers agree that results should be returned to participants, they acknowledge that they rarely do so. In this study, respondents identified many barriers that constrain them from returning results to study participants (Table 3). IRBs, CTSAs, and PRCs as well as agencies that fund research are well positioned to help overcome these barriers through changes in policies and practice that can encourage the results to be returned to participants. In August 2018, we returned a summary of the survey findings via e-mail to all respondents who requested the results.
